# The Biological Significance of Long noncoding RNAs Dysregulation and their Mechanism of Regulating Signaling Pathways in Cervical Cancer

**DOI:** 10.22088/IJMCM.BUMS.10.2.75

**Published:** 2021-09-01

**Authors:** Maryame Lamsisi, Lahcen Wakrim, Amal Bouziyane, Mustapha Benhessou, Mounia Oudghiri, Abdelilah Laraqui, Mohamed Elkarroumi, Mohammed Ennachit, Mohammed El Mzibri, Moulay Mustapha Ennaji

**Affiliations:** 1 *Team of Virology, Oncology and Medical Biotechnologies, * *Laboratory of Virology, Microbiology, Quality, and Biotechnologies/ ETB* *. Faculty of Science and Techniques Mohammedia, Hassan II University of Casablanca, Morocco.*; 2 *Laboratory of Virology, Pasteur Institute of Morocco. Casablanca, Morocco.*; 3 *University Mohammed VI of Health Science, Casablanca, Morocco.*; 4 *School of Medicine and Pharmacy, University Hassan II of Casablanca, Morocco.*; 5 *Immunology and Biodiversity laboratory, Faculty of Sciences Ain chock, Hassan II University of Casablanca, Morocco.*; 6 *Research and Biosafety Laboratory, Mohammed V Military Hospital, University Mohammed V of Rabat, Morocco.*; 7 *Biology and Medical Research Unit, CNESTEN, Rabat, Morocco.*

**Keywords:** Cervical cancer, human papillomavirus, long noncoding RNA, signaling pathways, gene regulation

## Abstract

Despite the remarkable decrease in cervical cancer incidence due to the availability of the HPV vaccine and implementation of screening programs for early detection in developed countries, this cancer remains a major health problem globally, especially in developing countries where most of the cases and mortality occur. Therefore, more understanding of molecular mechanisms of cervical cancer development might lead to the discovery of more effective diagnosis and treatment options. Research on long noncoding RNAs (lncRNAs) demonstrates the important roles of these molecules in many physiological processes and diseases, especially cancer. In the present review, we discussed the significance of lncRNAs altered expression in cervical cancer, highlighting their roles in regulating highly conserved signaling pathways, such as mitogen-activated protein kinase (MAPK), Wnt/β-catenin, Notch, and phosphatidylinositol 3-kinase/protein kinase B (PI3K/AKT) pathways and their association with the progression of cervical cancer in order to bring more insight and understanding of this disease and their potential implications in cancer diagnosis and therapy.

## Introduction

Worldwide, cervical cancer (CC) is a major public health issue, ranking the fourth most diagnosed cancer, and the second leading cause of cancer-related deaths in women (1). Clinical and epidemiological shreds of evidence reported that the occurrence of CC requires a prior persistent infection with human papillomavirus (HPV) (2). However, HPV infection alone is not sufficient and other cofactors including host genetic alterations and epigenetic modifications are needed for the progression from benign lesions to malignant tumors (3). The lack of accurate understanding of host factors and genetic background of this disease might explain the failure of current treatment options leading to high mortality rates.

The sequencing of the complete human genome by the human genome project in 2003 promised to offer more insights to our understanding of human physiology and resolving human genetic diseases including cancer (4). Thus, the encyclopedia of DNA elements (ENCODE) project that took over after human genome project completion, deciphered the obtained sequences and provided more in-depth data and analyzed the regulatory elements within the genome (5). Among the biggest discoveries of ENCODE is that the non-coding part of the genome which was described as junk DNA is mostly transcribed into functional RNA molecules, named non-coding RNAs (ncRNAs) (6). This part of the genome is not fully characterized despite the numerous studies on ncRNAs, providing an enormous field of genomics that is yet to be explored.

Several hypotheses are suggested regarding the role of ncRNAs, but their role in gene regulation is well discussed as they influence gene expression without DNA sequence alterations (7). ncRNAs are divided into 2 subclasses according to the length of the RNA molecule: small ncRNA (sncRNA) (20–200 nucleotides) and long ncRNA (lncRNA) (more than 200 nucleotides).

Emerging findings report that lncRNAs, with tissue-specific expression, are involved in diverse cellular and physiological pathways including cell differentiation, maintaining cellular homeostasis, regulation of the immune response to disease, differentiation, and DNA damage repair (8,9). During malignancy, aberrant expression of lncRNAs is reported in many cancers, suggesting their role in the modulation of the physiological and molecular changes occurring in the transformed cells (9). Evidence from previous researches indicates that lncRNAs mainly interact with proteins, RNA, and DNA and function at transcriptional, translational, and post-translational levels (10). Moreover, Khalil et al. have reported that more than 20% of lncRNAs bind to the polycomb repressive complex 2 (PRC2) and other chromatin modifiers suggesting that chromatin modification might be a common mechanism of lncRNAs action (11).

In cervical cancer, an increasing number of functional studies have reported that dysregulation of the expression of diverse lncRNAs is involved in the regulation of malignant progression. In fact, the abnormal expression patterns of lncRNAs often correlate with the development and progression of cancer and play a crucial role in cell proliferation, invasion, and metastasis (12–14). LncRNAs exert their functions in CC mainly through the regulation of gene expression, which appears to be mediated by different processes such as chromatin state modulation and RNA processing (15). In CC, a number of lncRNAs showed abnormal expressions, such as HOX antisense intergenic RNA (*HOTAIR*), plasmacytoma variant translocation 1 (*PVT1*), and growth arrest specific 5 (*GAS5*), which are associated with disease progression and poor prognosis (16–18). On another hand, growing interest is given to the role of lncRNAs in viral replication and pathogenesis supporting their involvement in the host-pathogen interaction and suggesting the initiation and promotion of associated diseases (19,20). In the present review, we discuss the significance of lncRNAs altered expression in CC, highlighting their roles in regulating highly conserved signaling pathways, such as mitogen-activated protein kinase (MAPK), Wnt/β-catenin, Notch, and phosphatidylinositol 3-kinase/protein kinase B (PI3K/AKT) pathways and their association with the progression of CC.

**The implication of lncRNAs in cancer progression**

Up to now, many lncRNAs have been reported in CC and are involved in cell proliferation, cell cycle, apoptosis, epithelial to mesenchymal transition (EMT), migration, and/or invasion, such as *GAS5*, *HOTAIR*, metastasis associated lung adenocarcinoma transcript 1 (*MALAT1*), small nucleolar RNA host gene 8 (*SNHG8*), long intergenic non-protein coding RNA 511 (*LINC00511*) and MAGI2 antisense RNA 3 (*MAGI2-AS3*) that were also widely considered as specific biomarkers for early diagnosis (21–26). Studies on the different mechanisms and interactions of lncRNAs with other genes and proteins that confirm the involvement of lncRNAs in CC development and progression are summarized in Table 1. 

Almost all of the lncRNAs studied in CC interfere with cell proliferation through direct or indirect interaction with cell cycle proteins and apoptosis pathways. Highly expressed C5orf66 antisense (*C5orf66-AS1*) in CC was reported to decrease the number of cells in the G1/G0 phase while increasing cell numbers in the G2/S phase. Moreover, overexpression of *C5orf66AS1* promoted the proliferation and affected apoptosis and cell cycle through adsorbing the regulator miR-637 (13). LncRNA NCK1-antisense 1 (*NCK1-AS1*) has been reported to promote cell proliferation and to induce cell cycle progression in CC by interacting with miR-6857 and affecting the cyclin-dependent kinase 1 pathway. *NCK1-AS1* induced elevated expression of cyclin dependent kinase (*CDK1/6*) by antagonizing miR-6857 and led to the control of the G1-S transition in CC cell lines (43,44).

Initially, *MAGI2-AS3* was reported to have tumor suppressive activities. However, Liu et al. have found that *MAGI2-AS3* up-regulated *CDK6* and enhanced cell proliferation in CC (24). This oncogenic treat has been reported in other recent studies confirming that *MAGI2-AS3* promotes other cancers types such as colorectal and gastric cancers (45,46). On the other hand, *GAS5* was reported as a tumor suppressor lncRNA. Its ectopic overexpression induced cell cycle arrest at G2/M checkpoint which is mediated by the inhibition of cyclin B1 and *CDK1* expression by *GAS5*. Elevated expression of *BAX *and suppression of *BCL-2* is also a consequence of *GAS5* overexpression, which ultimately induces apoptosis (22).

LncRNA *MALAT1* was previously reported to be highly expressed in CC cells, and was correlated with cancer progression and metastasis (47). *MALAT1* is over-expressed in CC, and regulates the expression of apoptosis-related genes such as caspase-3 and 8, *BAX*, *BCL-2*, and *BCLxL* (48). Recent data suggest that HPV E6/E7 and IL-6/STAT3 signaling pathways work synergistically to up-regulate the transcription of *MALAT1* in CC HeLa cells, suggesting the cooperation of the virus oncoproteins with cellular inflammatory signaling in CC development (49).

In vitro studies on CC cell lines, showed that *HOTAIR* plays a role in apoptosis as its knockdown decreased protein levels of anti-apoptotic BCL-2, while it increased protein levels of pro-apoptotic BAX, apoptotic protease activating factor (APAF), caspase-3, caspase-9, and poly ADP-ribose polymerase (PARP) (26). *SNHG8*, another oncogenic lncRNA, promotes cell proliferation and inhibits apoptosis by recruiting enhancer of zeste homolog 2 (EZH2) to induce the trimethylation of reversion inducing cysteine rich protein with kazal motifs (*RECK*) promoter and thus inhibiting its expression (23). In addition, *LINC00511* recruits transcription factor retinoid X receptor alpha (RXRA) to upregulate the expression of phospholipase D1 (*PLD1*), and its knockdown promotes autophagy and apoptosis (21).

**Table 1:** LncRNAs interactions and roles in cervical cancer

**Lnc** **RNA**	**Expression level**	**Interaction with**	**Mechanism**	**Biological** **process**	**Ref.**
***MALAT1***	Up	EMT genes	MALAT1 up-regulated Transcription factor snail and levels of β-catenin and Vimentin while downregulated E-cadherin and ZO-1	Invasion and Migration	(25)
***MAGI2-AS3***	Up	CDK6	MAGI2-AS3 up-regulated CDK6	Cell proliferation and cell cycle	(24)
***HAND2-AS1***	Down	ROCK1	HAND2-AS1inhibited the expression of ROCK1	Cell proliferation migration and invasion	(27)
	Down	C16orf74	HAND2-AS1 recruited transcription factor E2F4 to C16orf74 promoter and suppressed its expression	Cell proliferation, migration and invasion	(28)
***SOX2OT***	Depends on variants	SOX2	SOX2OT modulated CC progression via the regulation of SOX2	Cell proliferation migration and invasion	(29)
***SNHG16***	Up	SPI1/ PARP9 Axis	SNHG16 recruited SPI1 protein to promote transcription of PARP9.	Cell Proliferation, invasion and Cell Metastasis	(30)
***TUG1***	Up	PUM2	TUG1 enhanced the progression of CC by its interaction with PUM2.	Cell proliferation and migration	(31)
***MEG3***	Down	P-STAT3	MEG3 bound directly to P-STAT3 protein and induced its ubiquitination and degradation.	Cell proliferation, apoptosis	(32)
***LINC00511***	Up	RXRA/ PLD1	LINC00511 enriched RXRA to the promoter region of PLD1 and promoted its expression.	Cell proliferation, Apoptosis and tumor growth.	(21)
***MIR205HG***	Up	- SRSF1 - KRT17	Lnc-RNA MIR205HG regulated CC progression through KRT17 by binding with SRSF1	Cell proliferation, apoptosis and Migration	(33)
***lncOGFRP1***	Up	EMT and Apoptosis proteins	The depletion of lncOGFRP1 inhibited the expression of β‐catenin, Vimentin, N‐cadherin, SNAIL, Bcl‐2, cyclinA1, CDK2, and PCNA, and promoted the expression of E‐cadherin, Bax, p53, and caspase3	Cell proliferation, Cell cycle apoptosis and migration	(34)
***GPC3‐AS1***	Up	GPC3	ELK1 acts as the transcription activator of GPC3‐AS1 and GPC3	Cell proliferation and migration	(35)
***CRNDE***	Up	PUMA	CRNDE binds to PUMA to inhibit its expression.	Cell proliferation, apoptosis and Tumor growth	(36)
***LINC00052***	Down	STAT3	The mRNA and protein expression of STAT3 was downregulated after overexpressing LINC00052.	Cell proliferation, tumor growth, invasion and migratin	(37)
***GAS5***	Down	Cyclin B1 and CDK1	GAS5 induced Cell cycle arrest by reducing the expression of Cyclin B1 and CDK1	Cell proliferation, Cell cycle, Apoptosis, tumor growth, Invasion and migration	(22)
***SNHG8***	Up	EZH2 / RECK	SNHG8 bound to EZH2 and epigenetically inhibited RECK transcription in CC.	Cell proliferation and migration	(23)
***SNHG12***	Up	ERK/Slug	SNHG12 is modulated by human papillomavirus 16 E6/E7 and promoted CC progression via ERK/Slug pathway	Tumor growth and migration	(38)
***Lnc-CC3***	Up	Slug	Lnc-CC3 increased Slug expression, and reduced the expression of E-cadherin.	Migration and invasion	(39)
***ARAP1-AS1***	Up	c-Myc	ARAP1-AS1 might interact with PSF to release PTB, which accelerated IRES-driven translation of proto-oncogene c-Myc	Cell proliferation and migration	(40)
***LINP1***	Up	KLF2 and PRSS8	LINP1 scaffolded EZH2, LSD1 and DNMT1 to suppress KLF2 and PRSS8	Cell proliferation and apoptosis	(41)
***LINC02535***		PCBP2	LINC02535 interacted with PCBP2 to regulate DNA damage repair by stabilizing RRM1 mRNA in CC	Cell proliferation, migration and invasion,	(42)

ARAP1-AS1: ARAP1 Antisense RNA 1; C16orf74: Chromosome 16 Open Reading Frame 74; CC: Cervical cancer; CDK1: Cyclin Dependent Kinase 1; CDK6: Cyclin Dependent Kinase 6; ROCK1: Rho Associated Coiled-Coil Containing Protein Kinase 1; CRNDE: Colorectal Neoplasia Differentially Expressed; DNMT1: DNA Methyltransferase 1;E2F4: E2F Transcription Factor 4; EMT: Epithelial to Mesenchymal Transition; ERK: Extracellular‐signal‐regulated kinase; EZH2: Enhancer Of Zeste 2 Polycomb Repressive Complex 2 Subunit; GAS5: Growth Arrest Specific 5; GPC3: Glypican 3; GPC3‐AS1: GPC3 Antisense RNA 1; HAND2-AS1: HAND2 Antisense RNA 1; KLF2: Kruppel Like Factor 2; KRT17: Keratin 17; LINC00052: Long Intergenic Non-Protein Coding RNA 52; LINC00511: Long Intergenic Non-Protein Coding RNA 511; LINC02535: Long Intergenic Non-Protein Coding RNA 2535; LINP1: LncRNA In Non-Homologous End Joining Pathway 1; lncOGFRP1: Long noncoding RNA OGFRP1; LSD1: Lysine-specific demethylase 1; MAGI2-AS3: MAGI2 Antisense RNA 3; MALAT1: Metastasis Associated Lung Adenocarcinoma Transcript 1; MEG3: Maternally Expressed 3; MIR205HG: MIR205 Host Gene; PARP9: Poly(ADP-Ribose) Polymerase Family Member 9; PCBP2: Poly(RC) Binding Protein 2; PLD1: Phospholipase D1; PRSS8: Serine Protease 8; PSF: PTB-associated Splicing Factor; PTB: Polypyrimidine tract-binding protein; PUM2: Pumilio RNA Binding Family Member 2; PUMA: P53 upregulated modulator of apoptosis; RECK: Reversion Inducing Cysteine Rich Protein with Kazal Motifs; RRM1: Ribonucleotide Reductase Catalytic Subunit M1; RXRA: Retinoid X Receptor Alpha; SNHG12: Small Nucleolar RNA Host Gene 12; SNHG16: Small Nucleolar RNA Host Gene 16; SNHG8: Small Nucleolar RNA Host Gene 8; SOX2: SRY-Box Transcription Factor 2; SOX2OT: SOX2 Overlapping Transcript; SPI1: Spi-1 Proto-Oncogene; SRSF1: Serine and arginine Rich Splicing Factor 1; STAT3: Signal Transducer and Activator of Transcription 3; TUG1: Taurine Up-Regulated 1; ZO-1: Zonula occludens-1

**The role of lncRNAs in epithelial to mesen-chymal transition, invasion, and migration**

EMT is a cellular biological program that drives the transition of cells between adherent epithelial state to mesenchymal phenotypes. Epithelial cells undergo series of changes to acquire the characteristics of mesenchymal cells such as stemness, motility, invasiveness, and resistance to therapy, leading to an increased ability of transformation and migration to distant organs (50). Several studies have indicated that EMT, invasion, and migration are in part regulated by some lncRNAs. For instance, lncRNA SRY-box transcription factor 2 (SOX2) overlapping transcript (*SOX2OT*) contributed to cell proliferation, migration and invasion of CC cells via the regulation of -box SOX2 (29). *HOTAIR* interacted with key genes that regulate cell invasion and metastasis such as *STAT3*, β-catenin, vascular endothelial growth factor (*VEGF*), E-cadherin, matrix metalloproteinases (*MMP-9*), vimentin, snail, and twist, all of which are involved in EMT, invasion, and migration (51). Consistently, Lee et al. (2016) have investigated the expression levels of EMT related genes in vivo and found that β-catenin, N-cadherin, vimentin, snail, and twist were highly expressed in tumors overexpressing *HOTAIR* in comparison with the controls (26).

LncRNA-CTS may contribute to EMT, migration and invasion in CC cells through TGF-β1. In fact, lncRNA-CTS regulates *TGF-β1* via sponging miR-505, which in turn is responsible for the regulation of zinc finger E-box binding homeobox 2 (*ZEB2*) mRNA (52). *ZNF667-AS1* is a tumor suppressor lncRNA that also employs sponging microRNA mechanism to reduce tumor invasion and metastasis in CC by competitive binding to miR-93-3p, and thus upregulating *PEG3* (53). 

*GAS5-AS1* is another tumor suppressor that inhibits cell proliferation and metastasis of CC both *in vitro* and *in vivo* through increasing the expression of another tumor suppressor lncRNA, *GAS5*. *GAS5-AS1* appear to enhance the stability of *GAS5*, and thus increasing its expression, by reducing its N6-methyladenosine (m6A) modification (54). 

**Involvement of lncRNAs in signaling pathways**

Deregulated expression of lncRNAs is involved in the initiation and promotion of CC development, invasion, and metastasis through their interactions with several signaling pathways. Numerous lncRNAs, comprising among others *HOTAIR*, *MALAT1*, *GAS5*, *EMT*, and maternally expressed gene 3 (*MEG3*) are involved in conserved signaling pathways such as Wnt, MAPK, NOTCH, and PI3K/AKT pathways (Table 2). Altogether, they have been shown to be associated with various pathogenic processes such as tumor progression, invasion as well as therapeutic resistance, and have emerged as new diagnostic and prognostic biomarkers in CC (55).

**LncRNAs interfere with the Wnt signaling pathway in CC**

Wnt/β-catenin is a highly conserved signaling pathway that plays key roles in the development of cancer through modulating cell growth, cell regulation, and cell differentiation. Abnormal activation of the Wnt signaling pathway, which is the result of aberrant genetic and epigenetic regulation of its components, is linked to the progression of various types of cancers, including CC (84). As for every signaling pathway, Wnt pathway requires spatiotemporal regulation to maintain appropriate biological response and to prevent disease.

Several studies indicate that lncRNAs induce malignant behavior in CC by playing important roles in this regulation. For instance, lncRNA colon cancer associated transcript 1 (*CCAT-1*) promotes cell proliferation through inhibiting apoptosis in CC cells and *RP11-480I12.5* induces the EMT of CC through the Wnt/β-catenin pathway (56,60). In addition, lncRNA *ASB16* antisense RNA 1 (*ASB16-AS1*) acts as a sponge of miR-1305 to prevent its inhibitory effect on Wnt2 and enhance cell proliferation, migration, and invasion (65).

**Fig. 1:**
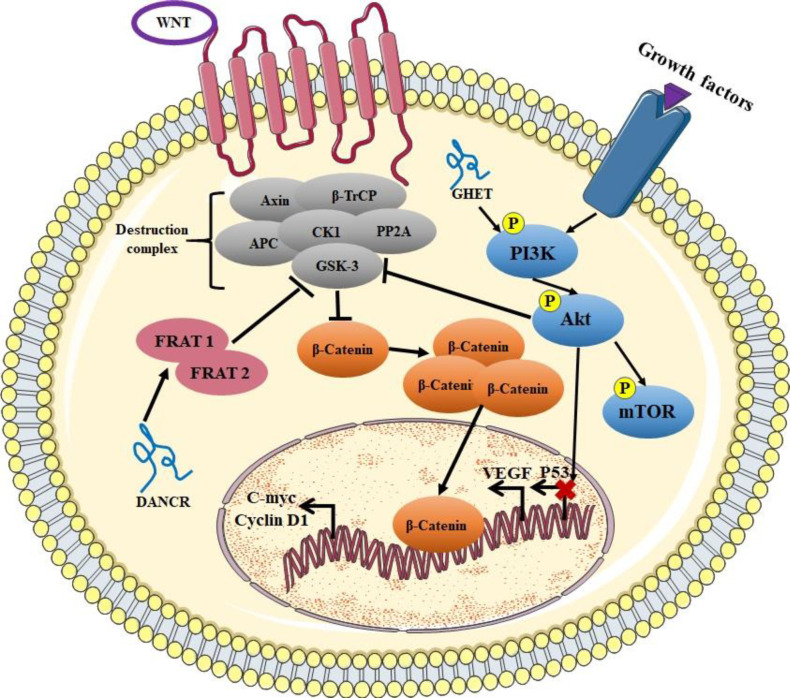
**Regulation of the Wnt/β-catenin signaling pathway and MAPK pathway by lncRNAs and the crosstalk between the pathways.** *DANCR* recruit *FRAT1* and *FRAT2* to negatively regulate GSK-3, which inhibits the accumulation of β-catenin and its translocation to the nucleus. LncRNA *GHET* positively regulates PI3K/AKT/mTOR pathway, which in turn targets GSK-3 and regulates Wnt/β-catenin pathway

*HOTAIR* is one of the most studied lncRNAs that is overexpressed in several cancers including CC, and is known by its role in modulating chromatin state by scaffolding the three components of the chromatin-modifying complex PRC2: EZH2, SUZ12, and embryonic ectoderm development (EED) and directs them to distant targeted loci, which consequently induces the H3K27 tri-methylation on promoters of specific genes (16)(85). Through a similar mechanism, *HOTAIR* appears to regulate the Wnt/β-catenin pathway as well. In fact, *HOTAIR* was found to recruit tet methylcytosine dioxygenase 1 (*TET1*) to induce methylation in the promoters of negative regulators of the Wnt/β-catenin pathway such as protocadherin 10 (*PCDH10*), *SOX17*, adherens junctions associated protein 1 (*AJAP1*), and *MAGI2*, to decrease their expression in HeLa cells (66) (Figure 1).

**Fig. 2:**
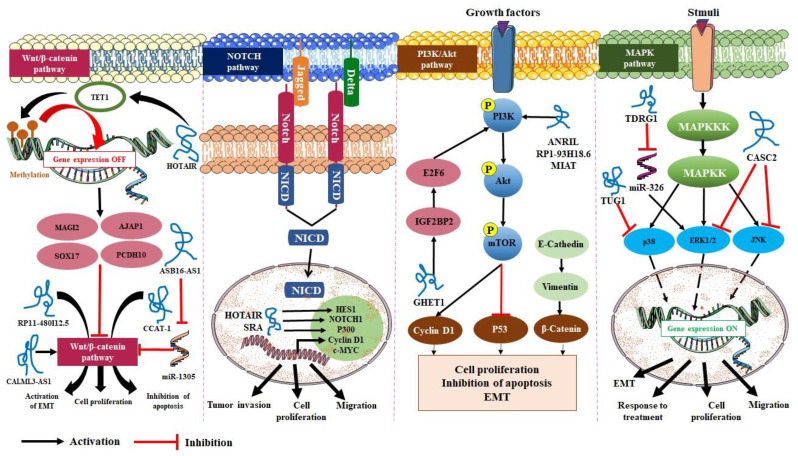
Involvement of lncRNAs in the regulation of conserved signaling pathways

In vitro downregulation of lncRNA cancer susceptibility 11 (*CASC11*) in HeLa cells, inhibits the activity of Wnt/β-catenin signaling pathway while overexpression of *CASC11* in CaSki cells significantly up-regulated the signaling activity, suggesting that *CASC11* was involved in the activation of Wnt/β-catenin signaling pathway (63). *CALML3* antisense RNA 1 (*CALML3-AS1*) is another overexpressed lncRNA in CC. The levels of the Wnt/β-catenin pathway-related proteins such as β-catenin, cyclin D1, and c-MYC were observed to be down-regulated due to *CALML3-AS1* knockdown in CC cells, suggesting that the activity of Wnt/β-catenin pathway is promoted by *CALML3-AS1*, which might be the mechanism by which *CALML3-AS1* promotes CC (59) (Figure 2). 

When Wnt is not expressed, cytoplasmic β-catenin is degraded by a protein complex composed of axin protein, adenomatous polyposis coli (APC), the E3-ubiquitin ligase β-TrCP, CK1, ser/thr kinases GSK-3 protein phosphatase 2A (PP2A), and glycogen synthase kinase 3 (GSK3). β-catenin degradation prevents its transfer to the nucleus, and thus repressing the transcription of Wnt targeted genes (86). 

**Table 2:** LncRNAs involved in regulating signaling pathways

**Pathway**	**LncRNAs involved**		**Ref.**
	**Activators**	**Inhibitors**	
**Wnt/β-catenin pathway**	*CCAT1; DANCR; BLACAT1; CALML3-AS1; RP11-480I12.5; SNHG7; PCAT6; CASC11; NNT-AS1; ASB16-AS1*	*DGCR5*	(56,57,66,67,58–65)
**PI3K/AKT/mTOR pathway**	*CRNDE; RP1-93H18.6; ANRIL; CCAT1; MFI2; NEAT1; MIAT*	*LINC00037 (DGCR5)*	(68–74)(75)
**NOTCH pathway**	*HOTAIR; SRA*	-	(26,76)
**NF** **‑** **κB Pathway**	*PVT1; NEAT1*	-	(77,78)
**MAPK Pathway**	*CASC2; MNX1-AS1; TUG1; TDRG1*	-	(16,79–82)
**JAK/STAT3**	*LINC00518*	-	(83)

ANRIL: antisense non-coding RNA in the INK4 locus; ASB16-AS1: ASB16 antisense RNA 1; BLACAT1: bladder cancer associated transcript 1; CALML3-AS1: CALML3 antisense RNA 1; CASC11: cancer susceptibility 11; CASC2: cancer susceptibility 2; CCAT1: colon cancer associated transcript 1; CRNDE: colorectal neoplasia differentially expressed; DANCR: differentiation antagonizing non-protein coding RNA; DGCR5: DiGeorge syndrome critical region gene 5; HOTAIR : HOX transcript antisense RNA ; JAK: janus kinase; LINC00037: long intergenic non-protein coding RNA 37; LINC00518: long intergenic non-protein coding RNA 518; MAPK: mitogen-activated protein kinase 1; MFI2: melanotransferrin 2; MIAT: myocardial infarction associated transcript; MNX1-AS1: MNX1 antisense RNA 1; NEAT1: nuclear paraspeckle assembly transcript 1; NF-κB: nuclear factor kappa B subunit 1; NNT-AS1: NNT antisense RNA 1; PCAT6: prostate cancer associated transcript 6; PVT1: plasmacytoma variant translocation 1; SNHG7: small nucleolar RNA host gene 7; SRA: steroid receptor RNA activator; STAT3: signal transducer and activator of transcription 3; TDRG1: testis development related 1; TUG1: taurine up-regulated 1.

Differentiation antagonizing non-protein coding RNA (*DANCR*) activates Wnt/β-catenin signaling pathway through positively regulating frequently rearranged in advanced T-cell lymphomas 1 (*FRAT1*) and *FRAT2* expressions which belong to the GSK-3-binding proteins family that inhibit GSK-3-mediated β-catenin phosphorylation and degradation, which allows β-catenin to reach the nucleus to regulate targeted genes expression (57) (Figure 1). Consistently, the findings of this study indicated that induced overexpression of *DANCR* enhanced the mRNA and protein expression levels of *c-MYC* and cyclin D1, which are targeted genes of the Wnt/β-catenin signaling pathway while knockdown of *DANCR* exhibited the opposite effect (57) (Figure 1).

**LncRNAs regulation of phosphatidylinositol 3-kinase (PI3K)/protein kinase B (AKT) pathway in CC**

PI3K is a member of the lipid kinases family. In the normal state of the cell, various extracellular factors, such as hormones, growth factors, and cytokines send signals to activate PI3K through the interaction with a phosphorylated tyrosine receptor. PI3K downstream cascade generates signals received by its targets, the most important one being the protein kinase B (AKT) that dominates the signal transduction of the PI3K pathway (87). Activation of AKT is a common phenomenon in human cancers leading to the promotion of cell proliferation (88). The entire PI3K/AKT signaling pathway plays key roles in regulating cell physiology and pathology, including apoptosis, cell proliferation, invasion, and metastasis (88). This pathway is abnormally activated in different tumors including CC (89). 

Among the many regulators of this pathway, lncRNAs are also involved, adding more complexity to these processes. Decreased expression of the a​ntisense non-coding RNA in the *INK4* locus (*ANRIL*) inhibits cell proliferation, migration, and invasion in CC. After the inhibition of *ANRIL*, the PI3K/AKT pathway was found to be inactivated in CC cells, which indicates that *ANRIL* might regulate CC progression through the PI3K/AKT pathway (70). In addition, overex-pressed *RP1-93H18.6* is an oncogenic lncRNA, its down-regulation resulted in the inhibition of cell proliferation and EMT in HeLa cells while promoting cell apoptosis via blocking the PI3K/AKT/mTOR signaling pathway (69). Moreover, lncRNA myocardial infarction associated transcript (*MIAT*) promotes CC and up-regulates PI3K, AKT, and mTOR levels, indicating its ability to activate PI3K/AKT/mTOR signaling pathway (74) (Figure 1).

LncRNA gastric carcinoma proliferation enhancing transcript 1 (*GHET1*) was found to regulate CC progression through modulating AKT/mTOR and its cross-talk with Wnt/β-catenin pathways (Figure 1) (90). mTOR is one of the downstream targets of the PI3K/AKT axis. The mTOR axis is up-regulated in CC, and is suggested as a therapeutic target for anti-CC drug development. Blocking mTOR has shown a significant effect in treating HPV-related oral cancer and CCs (91). The crosstalk between AKT/mTOR and Wnt/β-catenin has been demonstrated in many studies. In fact, p-AKT could induce the phosphorylation of Wnt protein receptor GSK3β, which as mentioned above, induces the accumulation and nuclear migration of β-catenin, leading to the activation of Wnt/β-catenin pathway (92) (Figure 2). However, the exact mechanism of action of lncRNAs in regulating this crosstalk in CC is not fully elucidated.

**LncRNAs and Notch signaling pathway in CC**

Notch signaling pathway plays an important role in different cellular processes such as cell proliferation and apoptosis. NOTCH signaling pathway has two main groups of ligands such as delta-like 1, 3, and 4 and jagged 1 and 2. The binding of these ligands to NOTCH receptors, such as NOTCH 1, 2, 3, and 4 induces the activation of the pathway (93). The activation of the pathways triggers NOTCH cleavage and release of activated NOTCH intracellular domain (NICD). NICD is then translocated into the nucleus, where it activates the transcription of its targeted genes, mainly hairy and enhancer of split-1 *(HES1), *cyclin D1*, *and* c-MYC.* Otherwise, NOTCH can initiate the activation of other signaling pathways such as PI3K-AKT (94).

The Crosstalk between lncRNAs and Notch pathway was found in several solid cancers. For instance, lncRNA *MIR22HG* inhibits gastric cancer development and progression through its negative interaction with NOTCH2 signaling (95). LncRNA *SNHG12* promotes the progression of osteosarcoma by sponging miR-195-5p, thereby up regulating *NOTCH2* (96). *GHET1* promotes prostate cancer progression through targeting KLF2 which activates the HIF-1alpha/NOTCH-1 pathway, and *MACC1-AS1* drives pancreatic cancer progression through activating PAX8/NOTCH1 signaling (97,98).

In CC, the NOTCH signaling pathway has a controversial role in alternating pro-oncogenic and tumor-suppressive roles (56). In vitro and in vivo studies showed that high levels of *HOTAIR* induce higher expression of *NOTCH1*, *HES1*, and *p300* in CC (26). Steroid receptor RNA activator (*SRA*) is a type of lncRNA which coordinates the functions of various transcription factors. *SRA *is related to the EMT and NOTCH signaling pathways, through which it induces in vitro tumor proliferation, migration and invasion (76) (Figure 2). These findings suggest that lncRNAs might promote CC through the NOTCH signaling pathway, representing an interesting way to deeply understand the complex role of this pathway in CC and its relation to lncRNAs.

**The role of lncRNAs in mitogen-activated protein kinase (MAPK) pathways in**

In its activated state, the MAPK phosphorylates its downstream targets in the nucleus and cytosol to regulate gene expression. There are three families of MAP kinases: JNKs (Jun amino-terminal kinases), ERKs (extracellular-signal-regulated kinases), and p38/SAPKs (stress-activated protein kinases). Numerous studies have shown that MAPK pathways play pivotal roles in CC (99), and numerous lncRNAs have been identified as regulators of the MAPK pathways in CC, through which they modulate cell proliferation, EMT, migration, and response to treatment (80–82).

LncRNA *CASC2* is reported to be down-regulated in CC, and acts as a tumor suppressor by inhibiting cell proliferation and migration. Overexpression of *CASC2* significantly inhibited the level of proteins of the MAPK pathway such as p-JNK and p-ERK1 *in vitro*, suggesting that *CASC2* might inhibit CC progression via negatively regulating the MAPK pathway (81). Jiang et al. demonstrated that testis development related gene 1 (*TDRG1*) sponged miR-326 to activate MAPK1, also known as ERK2, and thus suggested the miR-326/MAPK1 as a modulator of CC cell proliferation, migration, and invasion (79) (Figure 2).

In another study, lncRNA taurine up-regulated 1 (*TUG1*) controlled CC sensitivity to cisplatin through the MAPK pathway. *TUG1 *knockdown inhibited the proliferative rate but accelerated the apoptosis of cisplatin-induced CC cells (82). Both mRNA and protein levels of regulatory factor X7 (RFX7) were down-regulated by the *TUG1* knockdown. Indeed, knockdown of *RFX7 *could inhibit the proliferative rate and colony formation ability of CC cells. After cisplatin induction in CC cells, phosphorylated levels of *p38* and *JNK* increased, whereas *ERK1/2* expression decreased (82). *TUG1* knockdown could inhibit the proliferative rate and accelerate the apoptosis of CC cells by activating the MAPK pathway (82) (Figure 2). Zhang *et al.* analyzed the interaction between *HOTAIR* and STAT3. They identified a binding site for STAT3 in the promoter region of *HOTAIR* which is a GAS element. The genes containing GAS elements are regulated by STAT3, therefore, *HOTAIR* might be regulated by STAT3 as well. Moreover, they showed that *HOTAIR* and STAT3 affect synergically the aggressiveness of CC (100).

**Competing endogenous pathway of lncRNAs in CC**

It is widely accepted that gene regulation is more complex than previously expected, involving various regulators, enhancers, and/or transcription factors, acting *in* cis or *in* trans. Moreover, several studies have demonstrated that gene regulation is also mediated by microRNAs through complex mechanisms by which they interact with multiple networks. Since then, a growing interest was given to these microRNAs and their role in disease development, including cancer, which has been widely discussed and documented (101–103). Recently, several studies have reported that both coding and non-coding RNA molecules can regulate gene expression *in* cis and *in* trans by acting as sponges of microRNAs. These molecules, called competing endogenous RNAs (ceRNAs), represent a major group of gene regulators (104).

**Fig. 3:**
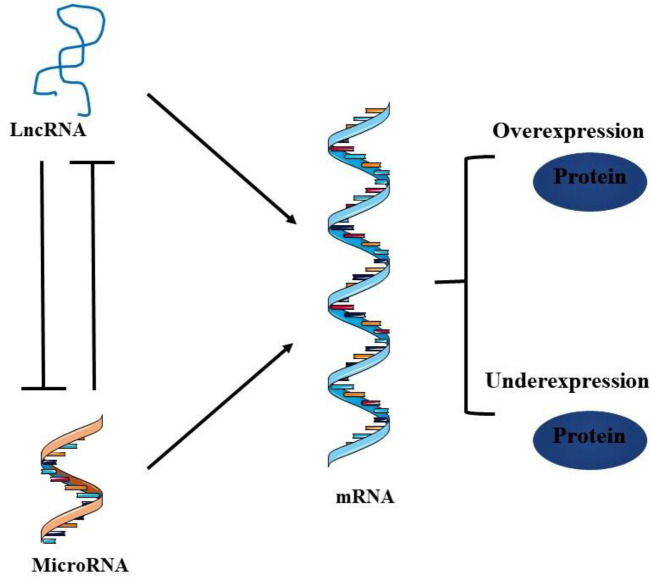
**Competing endogenous RNAs interaction. **There is a reciprocal negative regulation between LncRNAs and microRNAs, to both compete for mRNA binding sites. This competition leads eventually to gene expression and functional regulation

Intriguing relation is reported between lncRNAs and microRNAs; lncRNAs often act as molecular sponges or decoys to microRNAs and inactivate them. In turn, microRNAs have the ability to degrade lncRNAs. Together, lncRNAs and microRNAs can compete for binding sites on mRNAs (12,105) (Figure 3). Through this crosstalk between different RNA classes, lncRNAs regulate cancer progression and contribute to the regulation of cell proliferation, invasion, and migration in various cancer cells, including CC (12, 72, 73). Table 3 summarizes the main lncRNAs involved in CC development, their targeted microRNAs, and corresponding downstream dysregulated genes (12,105,106).

Of particular interest, most lncRNAs are up-regulated to sponge microRNAs and control cancer- development and progression. However, some of them are down-regulated and act as tumor suppressors. These include lncRNAs *STXBP5-AS1*, *TUSC8*, phosphatase and tensin homolog pseudogene 1 (*PTENP1*), and *CASC2* binding to miR-96-5p, miR‐641, miR-106b, and miR-21 respectively, to regulate the expression of *PTEN* (167–170). And lncRNA *miR503HG*, *WT1-AS*, *GAS5*, *FENDRR*, *LINC00173*, *MAGI2-AS3*, *MEG3*, and *ZNF667-AS1* that bind to miR-155, miR-203a-5p, miR-330-5p, miR-21, MiR-15a-5p/miR-15b-5p, miR-182-5p, miRNA-233, miR-7-5p, and miR-93-3p to regulate the expression of caspace-3, forkhead box N2 (FOXN2), P53, tubulin alpha 1a (*TUBA1A*), F-box/WD repeat-containing 7 (*FBXW7*), erythrocyte membrane protein band 4.1 like 3 (*EPB41L3*), *SCT1*, and paternally expressed gene (*PEG3*) that inhibit cell proliferation and induce apoptosis (53, 111, 119, 121, 122,143,146,162).

**Table 3:** Recent studies on ceRNA mechanism of LncRNAs in cervical cancer and downstream-targeted genes

**LncRNA**	**Expression level**	**Targeted miRNA**	**Downstream genes**	**Reference**
***SNHG16***	Up-regulated	miR-216-5p	*ZEB1*	(107)
	Up-regulated	miR-128	*GSPT1 *and *WNT3A*	(108)
***SNHG12***	Up-regulated	miR-125b	*STAT3*	(109)
***NEAT1***	Up-regulated	miR-133a	*SOX4*	(110)
	Up-regulated	miR-124	*NF-κB*	(78)
***MEG3***	Down-regulated	miR-7-5p	*STC1*	(111)
***MACC1-AS1***	Up-regulated	miR-34a	*CDK6*	(112)
***C5orf66-AS1***	Up-regulated	miR-637	*RING1*	(13)
***Linc00483***	Up-regulated	miR-508-3p	*RGS17*	(113)
***LINC01133***	Up-regulated	miR-4784	*AHDC1*	(114)
***LINC00152***	Up-regulated	miR-216b-5p	*HOXA1*	(115)
***lncRNA799***	Up-regulated	miR-454-3P	*TBL1XR1*	(116)
***LINC01503***	Up-regulated	miR-342-3p	*FXYD3*	(117)
***ZFPM2-AS1***	Up-regulated	miR-511-3p	*FGFR2*	(118)
***MAGI2-AS3***	Down-regulated	miRNA-233	*EPB41L3*	(119)
***MIR210HG***	Up-regulated	miR-503-5p	*TRAF4*	(120)
***LINC00173***	Down-regulated	miR-182-5p	*FBXW7*	(121)
***FENDRR***	Down-regulated	MiR-15a-5p/miR-15b-5p	*TUBA1A*	(122)
***CDKN2B-AS1***	Up-regulated	miR-181a-5p	*TGFβI*	(123)
***LINC01128***	Up-regulated	miR-383-5p	*SFN*	(124)
***NNT-AS1***	Up-regulated	miR-186	*HMGB1*	(125)
***PITPNA-AS1***	Up-regulated	miR-876-5p	*c-MET*	(126)
***ZNF667-AS1***	Down-regulated	miR-93-3p	*PEG3*	(53)
***TTN-AS1***	Up-regulated	miR-573	*E2F3*	(127)
***FOXP4-AS1***	Up-regulated	miR-136-5p	*CBX4*	(128)
***CASC9***	Up-regulated	miR-215	*TWIST2*	(129)
***LINC00473***	Up-regulated	miR- 34a	*ILF2*	(130)
***TP73AS1***	Up-regulated	microRNA607	*Cyclin D2*	(131)
	Up-regulated	microRNA-329-3p	*SMAD2*	(132)
	Up-regulated	miR‐329‐3p	*ARF1*	(133)
***TUG1***	Up-regulated	miR-138-5p	*SIRT1*	(134)
***DDN-AS1***	Up-regulated	miR-15a/ miR-16	*TCF3*	(135)
***EWSAT1***	Up-regulated	miR-330-5p	*CPEB4 *	(136)
***LINC00467***	Up-regulated	miR-107	*KIF23*	(137)
***SNHG20***	Up-regulated	miR-140-5p	*ADAM10*	(138)
***ATB***	Up-regulated	miR-144	*ITGA6*	(139)
***PCGEM1***	Up-regulated	miR-182	*FBXW11*	(140)
***CAR10***	Up-regulated	miR-125b-5p	*PDPK1*	(141)
***RP11-552M11.4***	Up-regulated	miR-3941	*ATF1*	(142)
***GAS5***	Down-regulated	miR-21	*STAT3*	(143)
***WT1-AS***	Down-regulated	miR-330-5p	*p53*	(144,145)
	Down-regulated	miR-203a-5p/	*FOXN2*	(146)
***HOTAIR***	Up-regulated	miR-148a	*HLA-G*	(147)
	Up-regulated	miR-23b	*MAPK1*	(16)
	Up-regulated	miR-143-3p	*BCL2*	(148)
	Up-regulated	miR206	*MKL1*	(149)
***H19***	Up-regulated	miR-138-5p	*SIRT1*	(150)
***DSCAM-AS1***	Up-regulated	mir-877-5p	*ATXN7L3*	(151)
***RHPN1-AS1***	Up-regulated	miR-299-3p	*FGF2*	(152)
***SBF2-AS1***	Up-regulated	miR-361-5p	*FOXM1*	(153)
***NR2F2-AS1***	Up-regulated	miR-4429	*MBD1*	(154)
***POU3F3***	Up-regulated	miR-127-5p	*FOXD1*	(155)
***DLG1-AS1***	Up-regulated	miR-107	*ZHX1*	(156)
***HCP5***	Up-regulated	microRNA-15a	*MACC1*	(157)
***TCONS_00026907***	Up-regulated	miR-143-5p	*ELK1*	(158)
***CRNDE***	Up-regulated	miR-183	*CCNB1*	(159)
***SPRY4-IT1***	Up-regulated	mir-101-3p	*ZEB1 *	(160)
***LINC01783***	Up-regulated	mir-199b-5p	*GBP1*	(161)
***miR503HG***	Down-regulated	miR-155	*Caspase-3*	(162)
***RHPN1-AS1***	Up-regulated	miR-299-3p	*FGF2*	(152)
***PVT1***	Up-regulated	miR-140-5p	*SMAD3*	(17)
***OIP5-AS1***	Up-regulated	miR-143-3p	*SMAD3*	(163)
	Up-regulated	miR-143-3p	*ITGA6*	(164)
***SNHG14***	Up-regulated	miR-206	*YWHAZ*	(165)
***UCA1***	Up-regulated	miR-493-5p	*HK2*	(166)
***STXBP5-AS1***	Down-regulated	miR-96-5p	*PTEN*	(167)
***CASC2***	Down-regulated	miR-21	*PTEN*	(168)
***TUSC8***	Down-regulated	miR-641	*PTEN*	(169)
***PTENP1 ***	Down-regulated	miR-106b	*PTEN*	(170)
	Down-regulated	miR-19b	*MTUS1*	(171)
***SOX21‐AS1***	Up-regulated	miR‐7	*VDAC1*	(172)
***TMPO-AS1***	Up-regulated	miR-577	*RAB14*	(173)
	Up-regulated	miR-143-3p	*ZEB1*	(174)
***TP73-AS1***	Up-regulated	miR-329-3p	*SMAD2*	(132)
	Up-regulated	miR-329-3p	*ARF1*	(133)
***LINC01535***	Up-regulated	miR‐214	*EZH2*	(175)
*** Linc00483***	Up-regulated	miR-508-3p	*RGS17*	(113)
***FOXD2-AS1***	Up-regulated	miR-760	*HDGF*	(176)
***PCAT6***	Up-regulated	miR-543	*ZEB1 *	(177)
***MIR205HG***	Up-regulated	miR-122e5p	*FOXP2*	(178)
***SNHG7***	Up-regulated	miR-485	*PAK4*	(179)
	Up-regulated	miR-485	*JUND*	(180)
***CCAT1***	Up-regulated	miR-181a-5p	*MMP14 *and* HB-EGF*	(181)
***HULC***	Up-regulated	miR-218	*TPD52*	(182)
***SOX21-AS1***	Up-regulated	miR-7	*VDAC1*	(172)
***BBOX1-AS1***	Up-regulated	miR-361-3p	*HOXC6*	(183)
***LncRNATP73‐AS1***	Up-regulated	miR‐329‐3p	*ARF1 *	(133)
***DLEU1***	Up-regulated	miR-381	*HOXA13*	(184)
***NOC2L‐4.1***	Up-regulated	miR‐630	*YAP1*	(185)
***LINC00319***	Up-regulated	miR-3127-5p	*RPP25*	(186)
***DLX6-AS1***	Up-regulated	miR-16-5p	*ARPP19*	(187)
***XIST***	Up-regulated	miR-200a	*FUS*	(188)
	Up-regulated	miR-889-3p	*SIX1*	(189)
	Up-regulated	MiR-140-5p	*ORC1*	(190)
***OIP5-AS1***	Up-regulated	miR-143-3p	*ROCK1*	(191)
***LINC00958***	Up-regulated	miR‐625‐5p	*LRRC8E*	(192)
	Up-regulated	miR- 5095	*RRM2*	(193)
***SNHG4***	Up-regulated	miR-148a-3p	*c-MET*	(194)
***RUSC1-AS1***	Up-regulated	miR-744	*BCL-2*	(195)
***NOC2L-4.1***	Up-regulated	miR-630	*YAP1*	(185)
***TINCR***	Up-regulated	miR-302	*Cyclin D1*	(196)
***TDRG1***	Up-regulated	miR326	*MAPK1*	(79)
	Up-regulated	miR-330-5p	*ELK1*	(197)
	Up-regulated	miR-214-5p	*SOX4*	(198)

ADAM10: A disintegrin and metalloproteinase 10; AHDC1: AT-hook DNA binding motif containing 1; ARF1: ADP ribosylation factor 1; ARPP19: cAMP regulated phosphoprotein 19; ATB: activated by transforming growth factor-β; ATF1: activating transcription factor 1; ATXN7L3: ataxin 7 like 3; BBOX1-AS1 : BBOX1 antisense RNA 1 ; BCL2: B-cell lymphoma 2; C5orf66-AS1: C5orf66 antisense RNA 1; CAR10: caspase recruitment domain family member 10; CASC2: cancer susceptibility 2; CASC9: cancer susceptibility 9; CBX4: chromobox 4; CCAT1: colon cancer associated transcript 1; CCNB1: cyclin B1; CDK6: cyclin dependent kinase 6; CDKN2B-AS1: CDKN2B antisense RNA 1; CPEB4: cytoplasmic polyadenylation element binding protein 4; CRNDE: colorectal neoplasia differentially expressed; DDN-AS1: DDN and PRKAG1 antisense RNA 1; DLEU1: deleted in lymphocytic leukemia 1; DLG1-AS1: DLG1 antisense RNA 1; DLX6-AS1: DLX6 antisense RNA 1; DSCAM-AS1: DSCAM antisense RNA 1; E2F3: E2F transcription factor 3; ELK1: ETS transcription factor ELK1; EPB41L3: erythrocyte membrane protein band 4.1 like 3; EWSAT1: Ewing sarcoma associated transcript 1; EZH2: enhancer of zeste homolog 2; FBXW11: F-box and WD repeat domain containing 11; FBXW7: F-box and WD repeat domain containing 7; FENDRR: FOXF1 adjacent non-coding developmental regulatory RNA; FGF2: fibroblast growth factor 2; FGFR2: fibroblast growth factor receptor 2; FOXD1: forkhead box D1; FOXD2-AS1: FOXD2 adjacent opposite strand RNA 1; FOXM1: forkhead box M1; FOXN2: forkhead box N2; FOXP2: forkhead box P2; FOXP4-AS1: FOXP4 antisense RNA 1; FUS: fused in sarcoma; FXYD3: FXYD domain containing ion transport regulator 3; GAS5: growth arrest specific 5; GBP1: guanylate binding protein 1; GSPT1: G1 to S phase transition 1; WNT3A: Wnt family member 3A; H19: H19 imprinted maternally expressed transcript; HB-EGF: heparin binding EGF like growth factor; HCP5: HLA complex P5; HDGF: heparin binding growth factor; HK2: hexokinase 2; HLA-G: major histocompatibility Ccomplex class I, G; HMGB1: high mobility group box 1; HOTAIR: HOX transcript antisense RNA; HOXA1: Homeobox A1; HOXA13: homeobox A13; HOXC6: homeobox C6; HULC: hepatocellular carcinoma up-regulated long non-coding RNA; ILF2: interleukin enhancer binding factor 2; ITGA6: integrin subunit alpha 6; JUND: jun D proto-oncogene subunit; KIF23 : kinesin family member 23; LINC: long intergenic non-coding RNA; TP73‐AS1: TP73 antisense RNA 1; LRRC8E: leucine rich repeat containing 8 VRAC subunit E; MACC1: MET transcriptional regulator MACC1; MACC1-AS1: MACC1 antisense RNA 1; MAGI2-AS3: MAGI2 antisense RNA 3; MAPK1: mitogen-activated protein kinase 1; MBD1: methyl-CpG binding domain protein 1; MEG3: maternally expressed gene 3; MIR205HG: MIR205 host gene; MKL1: megakaryoblastic leukemia 1; MMP14: matrix metallopeptidase 14; MTUS1: microtubule associated scaffold protein 1; NEAT1: nuclear paraspeckle assembly transcript 1; NF-κB: nuclear factor kappa B subunit 1; NNT-AS1: NNT antisense RNA 1; NOC2L-4.1: long noncoding RNA NOC2L-4.1; NR2F2-AS1: NR2F2 antisense RNA 1; OIP5-AS1: OIP5 antisense RNA 1; ORC1: origin recognition complex subunit 1; PAK4: P21 activated kinase 4; PCAT6: prostate cancer associated transcript 6; PCGEM1: prostate-specific transcript; PDPK1: 3-phosphoinositide dependent Protein kinase 1; PEG3: paternally expressed 3; PITPNA-AS1: PITPNA antisense RNA 1; POU3F3 : POU class 3 homeobox 3; PTEN : phosphatase and tensin homolog; PTENP1 : phosphatase and tensin homolog pseudogene 1; PVT1: plasmacytoma variant translocation 1; RAB14: member RAS oncogene family; RGS17: regulator of G protein signaling 17; RHPN1-AS1: RHPN1 antisense RNA 1; RING1: ring finger protein 1; ROCK1: rho associated coiled-coil containing protein kinase 1; RPP25: ribonuclease P and MRP subunit P25; RRM2: ribonucleotide reductase regulatory subunit M2; RUSC1-AS1: RUSC1 antisense RNA 1; SBF2-AS1: SBF2 antisense RNA 1; STC1: stanniocalcin 1. SFN: stratifin; SIRT1: sirtuin 1; SIX1: sine oculis  homeobox 1; SMAD2: SMAD family member 2; SMAD3: SMAD family mmber 3; SNHG: small nucleolar RNA host gene; SOX21-AS1: SOX21 antisense RNA 1; SOX4: SRY-box transcription factor 4; SPRY4-IT1: SPRY4 intronic transcript 1; STAT3: signal transducer and activator of transcription 3; STXBP5-AS1: STXBP5 antisense RNA 1; TBL1XR1: TBL1X receptor 1; TCF3: transcription factor 3; TDRG1: testis development related 1; TGFβI: transforming growth factor beta 1; TINCR: TINCR ubiquitin domain containing; TMPO-AS1: TMPO antisense RNA 1; TP73-AS1: TP73 antisense RNA 1; TPD52: tumor protein D52; TRAF4: TNF receptor associated factor 4; TTN-AS1: TTN antisense RNA 1; TUBA1A: tubulin alpha 1a; TUG1: taurine up-regulated 1; TUSC8: tumor suppressor candidate 8; TWIST2: twist family BHLH transcription factor 2; UCA1: urothelial cancer associated 1; VDAC1: voltage dependent anion channel 1; WT1-AS: WT1 antisense RNA 1; XIST: X inactive specific transcript; YAP1: Yes1 associated transcriptional regulator; YWHAZ: tyrosine 3-monooxygenase/tryptophan 5-monooxygenase activation protein zeta; ZEB1: zinc finger E-box binding homeobox 1; ZFPM2-AS1: ZFPM2 antisense RNA 1; ZHX1: zinc fingers and homeoboxes 1; ZNF667-AS1: ZNF667 antisense RNA 1

The regulation of microRNAs by lncRNAs was also investigated for a better understanding of the treatment outcome in patients with CC. For instance, Feng et al. have shown that TNF-α treatment induced overexpression of lncRNA *LOC105374902*, which acts as a ceRNA for miR-1285-3p to promote the expression of ribosomal protein L14 (*RPL14*), and thereby promoting the migration, invasion, and EMT of CC cells (199). Overexpression of lncRNA prostate cancer associated transcript 6 (*PCAT6*) down-regulated the expression of miR-543 in CC cells, thereby enhanced the level of zinc finger E-box-binding homeobox 1 (ZEB1), playing a key role in chemo-resistance of CC cells to cisplatin, and consequently promoting cell proliferation and metastasis (177).

**LncRNAs interaction with HPV in CC**

**Fig. 4:**
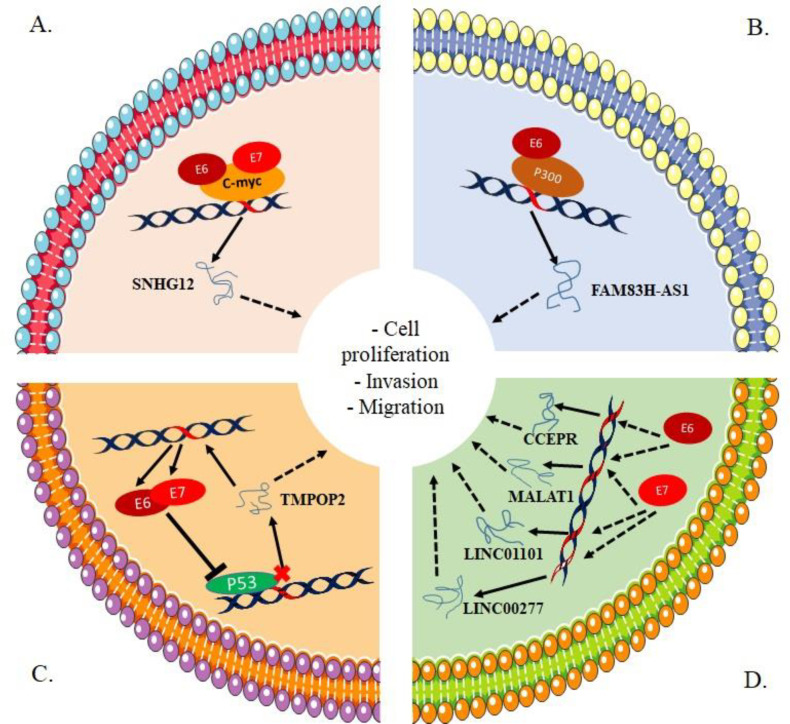
**LncRNAs and their interaction with HPV viral proteins in cervical cancer.** A) viral oncoproteins E6 and E7 recruit transcriptional factor c-MYC to induce the expression of lncRNA *SNHG12*; B) E6 enhances the expression of lncRNA *FAM38H-AS* through a mechanism involving P300; C) E6 and E7 form a regulatory feedback loop with lncRNA *TMPOP2*, where E6 and E7 inhibit P53 and its inhibitory effect on *TMPOP2 *expression, and *TMPOP2* induces the expression of E6 and E7, to promote CC; D: HPV viral proteins E6 and/or E7 mediate the overexpression of lncRNAs *MALAT1*, CCER, *LINC01101* and *LINC00277* in CC

HPV infection is a key event prior to CC development. Since HPV infection interferes with cellular mechanisms to induce aberrant cell proliferation, it was hypothesized that HPV interacts with lncRNAs in CC as well. Several studies demonstrated that lncRNAs are dysregulated in HPV positive cells and tissues (38, 200–203). This dysregulation is mainly mediated by HPV viral oncoproteins E6 and/or E7 (Figure 4).

Yang et al. reported significant change in lncRNAs expression patterns in HPV positive CC cell lines in comparison with HPV negative cells. They also found that these altered lncRNAs interacted with mRNAs that appear to play key roles in key cellular processes such as DNA repair, cell death, response to stimuli among others, all of which being involved in HPV related oncogenesis (203). In Barr et al. study, RNA high-throughput sequencing (RNA-seq) analysis indicated that the expression of host lncRNAs was altered in primary human foreskin keratinocytes cells (HEK) after infection with HPV16* E6* oncogene. The study showed that 151 lncRNAs were up-regulated and 100 were down-regulated. In addition, altered expression of some lncRNAs was observed between pre-malignant and cancerous cervical cells (200). Of particular interest, they further evaluated the expression of *FAM83H-AS1* lncRNA in primary human cervical keratinocytes (HCK) infected with HPV 16 whole-genome and they found higher expression levels of *FAM83H-AS1* in comparison with controls (200). *FAM83H-AS1* expression was also increased in HPV 16 positive cervical cell lines (CaSki, W12/201402, W12/20863), and decreased in HPV negative CC cell line (C-33A) in comparison with HCK cells (200). They demonstrated in the same study that *FAM83H-AS1* upregulation by HPV 16 is mediated specifically by E6 in a mechanism that does not involve its major downstream target p53. Instead, E6 regulates *FAM83H-AS1* through p300 (200).

In another study, HPV16 E6 oncogene-induced lncRNA cervical carcinoma expressed PCNA regulatory (*CCEPR*) expression. Both HFK cells expressing HPV *E6/E7* and HPV positive CC cells (CaSki) expressed higher levels of *CCEPR*, suggesting the involvement of HPV in increasing *CCEPR* levels in CC. Moreover, *CCEPR* overexpression induced by HPV16 E6 was reported to occur in a p53 independent manner (204).

Microarray analysis showed that 3626 lncRNAs were aberrantly expressed in HPV positive cervical squamous cell carcinoma samples versus HPV negative normal controls. Among them, 2077 lncRNAs were upregulated and 1549 lncRNAs were downregulated. Further qPCR analysis confirmed the overexpression of *SNHG12*, *MALAT1*, *HCG11*, colorectal neoplasia differentially expressed (*CRNDE*), and *PVT1* (38). Lai et al. showed also that *SNHG12* expression is closely linked to the expression of HPV16 *E6* or *E7*; *SNHG12* expression was down-regulated in cells not expressing HPV16 *E6* or *E7* and up-regulated in cells overexpressing HPV16 *E6* or *E7*, suggesting that HPV16 oncoproteins E6 and E7 might regulate the expression of *SNHG12 *lncRNA through the modulation of *c-MYC* (38). 

In *E7*-siRNA transfected HeLa cells, microarray analysis showed that the expression of 15387 RNA species was modified in comparison with controls; among them were 731 lncRNAs and 203 lincRNAs indicating that HPV18 *E7* is involved in dysregulating of the expression of RNAs. Among the most dysregulated lincRNAs following E7 depletion,, *LINC01101* and *LINC00277* were particularly increased, which was further confirmed by qPCR analysis. In clinical samples of HPV positive CC patients, *LINC01101* and *LINC00277* expression was decreased in precancerous and cancerous lesions and their reduced expression correlated with high- risk HPV infections including HPV16 and HPV18 (205).

He et al. found that HPV16/18 proteins E6 and E7 promoted the expression of lncRNA  thymopoietin pseudogene 2 (*TMPOP2*) in CC cells in a mechanism involving p53. Precisely, they found that p53 represses the expression of *TMPOP2* by direct binding to its promoter. *TMPOP2* in turn regulates the expression of HPV16/18 *E6/E7* and enhances their mRNA and protein level at a post-transcriptional level, suggesting that HPV16/18 E6/E7 along with lncRNA *TMPOP2* form a positive regulatory loop to regulate gene expression in CC in a synergic manner (206).

*MALAT1* was significantly overexpressed in high-risk HPV positive CC cells and tissues in comparison with normal controls and promoted cell proliferation and invasion. In addition, knockdown of HPV *E6/E7* inhibited *MALAT1* expression in CasKi cells. In clinical samples, *MALAT1 *was expressed in 30% of HPV-positive normal cervical cells and 60% of HPV-positive cervical lesions, while no expression of *MALAT1* was identified in HPV-negative normal cervical squamous cells (47).

Controversially, cells transfected with HPV16* E7* expressed lower levels of *HOTAIR*, which was described in many studies cited above as an oncogene. Lower expressions of neuropilin 2 (NRP2) and P53 as well as a higher level of miR331-3p were also reported in cells transfected with HPV16* E7*, which induced cell growth and inhibited apoptosis. Consistently with these findings, normal HPV positive cervical tissues also showed a reduced level of *HOTAIR* and *NRP2* in comparison with HPV negative normal cervical tissues (202). The interaction of lncRNAs with HPV infection has also diagnosis and therapeutic significance. For instance, LncRNA oncogene-induced senescence 1 (*OIS1*) was down-regulated in tissues and sera from HPV-positive patients with cervical squamous cell carcinoma and no significant differences were observed between HPV-negative patients and healthy controls. Consistently, *OIS1* expression levels were lower in HPV-positive cancer cell lines in comparison with that in HPV-negative cancer cell lines, while no significant differences were found between HPVpositive and HPV-negative normal cell lines. In addition, ROC curve analysis demonstrated that *OIS1* could potentially be used as a diagnostic marker for HPV positive but not for HPV negative cervical squamous cell carcinoma (207). Interestingly, it was found that damage induced noncoding (DINO) lncRNA could restore the function of *TP53 *in CC. The reactivation of *TP53 *by *DINO* increases the vulnerability of CC to standard chemotherapeutics as well as biguanide compounds that cause metabolic stress, which suggests that this lncRNA could be used as a therapeutic alternative to the existing unsuccessful approaches (201).

## Conclusion

The field of research on lncRNAs is growing each day with newly discovered molecules and new roles and mechanisms of already characterized ones; which provides a large variety of potential clinical applications. LncRNAs function either by direct interaction and inhibition of targeted signaling molecules or indirectly by binding other intermediate molecules such as mRNAs, proteins and microRNAs to alter their regulatory functions. 

In CC, a number of lncRNAs such as *HOTAIR*, *PVT1*, *MALAT1*, and *GAS5*, which are associated with disease progression and prognosis, showed abnormal expressions. They are also involved in the regulation of conserved signaling pathways, such as the Wnt/β-catenin, NOTCH, PI3k/AKT and MAPK pathways. In addition, most lncRNAs are up-regulated to sponge microRNAs and promote cancer development and progression, while, some of them are down-regulated and act as tumor suppressors; these include lncRNAs *STXBP5-AS1*, *TUSC8*, *PTENP1*, and *CASC2*.

Giving the unavailability of effective treatments for most advanced CCs, lncRNAs diversity in terms of roles and mechanisms provides another set of opportunities. However, lncRNAs occupy several cellular localizations and exert their regulatory functions in a wide range of cellular and pathological contexts. A single lncRNA might also possess different binding sites, and can function through different mechanisms depending on the cellular context. Therefore, more thorough studies are needed to identify key binding sites and to uncover their exact mechanism of action in HPV infection and CC progression to provide precise and targeted options for clinical applications. In addition, tissue specificity and the correlation of lncRNA expression to malignant phenotypes and also to viral infection provides a large field of biomarker research. Thus, more studies on the clinical applications of lncRNAs are required for new targeted therapy approaches and biomarker discoveries.
